# Validation of a New Cognitive Screening Method for Stroke Patients

**DOI:** 10.1155/2019/2943603

**Published:** 2019-11-06

**Authors:** Katri Saar, Hannu Nyrkkö, Asko Tolvanen, Pekka Kuikka, Erja Poutiainen, Tuija Aro

**Affiliations:** ^1^South Savo Social and Health Care and Authority, Finland; ^2^Kruunupuisto Punkaharju Rehabilitation Center, Finland; ^3^University of Jyväskylä, Department of Psychology, Finland; ^4^Neuroarviot Ltd., Finland; ^5^Rehabilitation Foundation, Finland

## Abstract

**Objective:**

Two million adults under fifty years of age have a cerebral stroke every year worldwide. Neuropsychological assessment is the best way to identify poststroke cognitive dysfunction, but it is often time-consuming and can be tiring for the patient, and hospitals vary in their availability of neuropsychological expertise. A valid and reliable cognitive screening method could be advantageous in identifying patients who need comprehensive neuropsychological examination. Our purpose in this study was to validate a newly developed cognitive screening method as an identifier of cognitive dysfunction after stroke in working-aged patients.

**Methods:**

We analyzed new cognitive screening method concurrent validity by comparing it in two groups formed on the basis of a comprehensive neuropsychological examination for 77 stroke patients. We identified the best balance of sensitivity and specificity by using receiver operating characteristic curve analysis and investigated the impact of the sociodemographic variables to the screening method total score variation.

**Results:**

We found a significant correlation between the method's total score and performance in neuropsychological examination. The cognitive method's internal consistency was strong; Cronbach's alpha for all items was .818. The best balance of sensitivity (88%) and specificity (50%) was found at a total score cut point of 138. Subjects' age and length of education were each responsible for 10% of total score variation.

**Conclusions:**

This study shows promising results for this new cognitive screening tool's ability to identify poststroke cognitive decline and patients' need for further detailed neuropsychological examination.

## 1. Introduction

Every year, about two million adults under fifty years of age worldwide suffer from a stroke. Studies have shown that the prevalence of cognitive problems varies from 40% to over 90% depending on the definition of cognitive dysfunction [1, 2]. Cognitive decline is a proven negative predictor for returning to work or managing prestroke activities [3, 4]. Early identification and recognition of cognitive deficits caused by stroke is important for organizing adequate rehabilitation programs and for preventing and delaying further poststroke symptoms [5]. Cognitive dysfunction may stay unrecognized and become evident only after the patient returns to social and occupational activities, which might cause significant social and emotional distress [6, 7].

Routine cognitive screening for poststroke patients would help clinicians and patients to detect and diagnose cognitive deficits [8]. Neuropsychological assessment is the best way to identify poststroke cognitive dysfunction [1, 9], but it takes time and is tiring for the patient, especially at the acute stage of stroke [10]. There are also differences between and within hospitals in the availability of neuropsychological assessment [11]. The aim of the present study is to develop a brief screening method that would be easy to administer and interpret by nonneuropsychological health care professionals and would serve in identifying particularly working-aged patients in need of more acute neuropsychological examination and aid the patients' rehabilitation.

Poststroke cognitive capacity is commonly assessed using screening tests such as the Mini-Mental-State Examination (MMSE) [12], which was developed especially for dementia patients and has shown to be inadequate in assessing stroke patients' cognitive functions [13]. Cognitive Linguistic Quick Test [14] consists of ten tasks, half of which have minimal language demands, and therefore is suitable for individuals with linguistic problems. There seems to be, however, lack of validation study against comprehensive neuropsychological study or ability to detect poststroke cognitive dysfunction. Wechsler's Adult Intelligence Scale (WAIS) [15] is commonly used in neuropsychological assessment and has also proven to be insufficient for detecting poststroke cognitive dysfunction when used as a single method [1]. Montreal Cognitive Assessment (MoCA) [16] is another frequently used neuropsychological method for poststroke cognitive evaluation. It has proven to be a more appropriate method than MMSE [17, 18], but it has a low specificity compared to comprehensive neuropsychological test batteries [19], and relative insensitivity to a single nonmemory domain impairment of the MoCA has been reported [18]. The Cambridge Cognition (CAMCOG) examination [20] has also been used, but it has been criticized for being too time-consuming [21] and difficult to administer [22].

There is no consensus on which test should be used to assess poststroke cognitive dysfunction [1]. A good screening test should be easy and quick to use, sensitive, and specific enough to detect real cognitive problems instead of false positive dysfunction [23]. Attention [24], working memory [1], information processing speed [25], and executive functions [24] are shown to be especially dysfunctional cognitive areas after stroke. However, because deficits may occur in every cognitive domain [26], screening should take into account each of the cognitive domains [27]. The purpose of this study is to test the reliability and validity of a recently developed method: Cognitive Screening Method for Stroke Patients (CoMet) (Saar, unpublished; developed in project *Stroke Patients Successful Returning to Work*, 2011–2015). Our specific research questions are as follows:

1. Does the screening method show good content validity when compared to performance in neuropsychological assessment?
2. Are the CoMet subscales internally consistent?
3. At which CoMet total score would be found the best balance of sensitivity and specificity?
4. Do sociodemographic variables (age, gender, and educational level) have an impact on the CoMet total score variation?

## 2. Methods

### 2.1. Participants and Procedures

Participants were stroke patients who were referred to the Kruunupuisto Punkaharju Rehabilitation Center by their hospital's project coordinators approximately two weeks to two months after having a stroke. Neurologists evaluated each patient using the following criteria: the patient fit the criteria of the research study if he/she was under 69 years of age, was employed at the time of the onset of stroke, and had the opportunity to return to work after sick leave. Stroke was confirmed by diagnostic brain imaging, and if the results of imaging remained unclear, neurological assessment was performed. Because one of the aims of the research project was to study patients' ability to return to work after a stroke, patients on parental leave, nursing leave, or job alternation leave were excluded from the study. Patients with serious degenerative illnesses or severe stroke-related disabilities causing clinically estimated inability to return to work were excluded from the study. Furthermore, patients suffering a transient ischemic attack (TIA), multiple sclerosis (MS), Parkinson's disease, central nervous system infection, or severe brain injury or undergoing acute cancer treatment, hip replacement, or exoskeleton operation within two months, as well as patients with severe depression, psychotic disorder, or alcohol or psychoactive drug addiction, were excluded from the study.

Two of the 79 originally referred patients were excluded from the study due to not meeting the criteria. One was not employed at the time of stroke, and the other had previous head trauma. Mean age of the 77 subjects included in the study was 53 years (SD = 6.9, range 34–64). Mean education in years was 12.75 (SD = 3.1, range 7–21). The time between the stroke and rehabilitation assessment (see description below) at Kruunupuisto was an average of 51 days. The most common etiology was an infarct (see Table 1). Damage on the right side of the brain accounted for 27 patients, while damage on the left side accounted for 41. Nine of the subjects had bilateral damage. Of the 41 individuals with a left side damage, language dysfunction including aphasia was found from 18 patients. Of these 13 were mild, one was moderate and four were severe as assessed by qualified and experienced clinical neuropsychologist. All participants had Finnish as their mother tongue, and the assessments were performed in Finnish.

The patients were provided with a three-day assessment period at Kruunupuisto Punkaharju Rehabilitation Center. All patients underwent a comprehensive neuropsychological assessment administered by an experienced neuropsychologist. The assessment included CoMet. Taking into consideration subjects' assumable poststroke exhaustion, comprehensive neuropsychological assessment was divided into two different days. The participation was voluntary, and the participants gave written informed consent before participating. The Research Ethics Committee of the Northern Savo Hospital District approved the study and consent procedure.

### 2.2. Measures

#### 2.2.1. Neuropsychological Examination

The neuropsychological test battery covered the following cognitive domains: basic reading and mathematical skills, language, memory, visuospatial processing, executive functions and attention, and visuomotor and finger speed. The patients were divided into two subgroups based on their performances in the neuropsychological tests. A neuropsychologist evaluated subjects' performance in neuropsychological tests using national reference values and also took into account subjects' primary performance level estimated by each subject's educational history and cognitive performance. Subjects with no neuropsychological deficits by neuropsychologist's clinical judgement formed Group 0 and those with at least mild cognitive deficits belonged to Group 1.

*Basic reading and mathematical skills* were evaluated with a text reading task comprising a meaningful short story consisting of four sentences. Mathematical skills were assessed with a task comprising eight simple paper-and-pencil arithmetic operations [28].

*Language* functions were assessed with a modified Token Test [29]. Word fluency was assessed with a semantic category test (subject asked to name as many animals as possible in one minute) and with the Boston Diagnostic Aphasia Examination (BDAE) for visual naming [30, 31]. Similarities and information, two subtests from Wechsler's Adult Intelligence Scale-III (WAIS-III) [16], were also used.

*Memory* tests covered general and delayed memory and also working memory. General and delayed memory were assessed with Logical Memory I and II, Paired Associates, and Wordlist subtests of the Wechsler Memory Scale-III (WMS-III) [32] and with the Benton Visual Retention Test (BVRT) [33]. Working memory was assessed with the Digit Span subtest of the WAIS-III [15] and heterogeneous and homogeneous interference tasks [28].

*Visuospatial processing* skills were assessed with the Block Design subtest of the WAIS-III [15], the Figure Copying subtest from the Consortium to Establish a Registry for Alzheimer's Disease (CERAD) test battery [34], and the Clock test, in which a subject must draw hands on and recognize accurate time from four dials. Line search tasks [35], in which subjects need to identify parallel lines from different sides of the hemisphere, and the Line Bisection Test [36] were also used.

*Executive functions and attention* were assessed with the Wisconsin Card Sorting Test (WCST) [37] and the Tower Test of the Delis-Kaplan Executive Function System (D-KEFS) battery [38] or the Tower of Hanoi Test [39]. Both tower tests are goal-structured problem-solving tasks. In addition, parts four and five of the Frontal Assessment Battery (FAB) [40] and part B of the Trail Making Test (TMT) [41] were used to assess sensitivity to interference and impulse control. The Bourdon-Wiersma test [42] was used to assess visual perception and vigilance, as was a word fluency task with phonemic category (subject needs to produce as many words in given category as he is able within one minute). Additionally, modified Stroop test [43], a rapid color naming and interference task, was performed only if the subject was able to name colors in earlier performed BDAE visual naming test [30].

*Visuomotor and finger speed* were tested using the Coding subtest of WAIS-III [15], part A of TMT [41], and the tapping device, in which subjects must tap with a thumb as many times as possible in 10 seconds. These were used to examine motor function, specifically motor speed and lateralized coordination.

#### 2.2.2. Using CoMet

This evaluation can be administered in approximately 20 minutes. It consists of 12 parts (see Table 2) chosen to emphasize attention, working memory, processing speed, and executive function.

### 2.3. Data Analysis

First, correlation analysis was used to investigate the connection between the CoMet total scores of groups with and without neuropsychological dysfunction. Our purpose was to analyze whether the CoMet total score could differentiate between patients showing at least mild cognitive dysfunction and those without or with minor problems in the clinical neuropsychological assessment. Second, we assessed the internal consistency of CoMet with Cronbach's alpha. Item-to-total correlations were also calculated to better describe the CoMet consistency. Third, we performed a nonparametric receiver operating characteristic (ROC) curve analysis to analyze the sensitivity and specificity of the screening method. Fourth, we used linear regression analysis to analyze whether sociodemographic variables (age, gender, and education) had an impact on the CoMet total score variation.

## 3. Results

The preliminary analyses indicated that the Word Fluency subtest gave too much weight to the total score, and thus, its scoring was modified. The highest possible score of the task was changed to 16, and scores above that were altered to 16. After the modification, the CoMet total score varied between 36 and 147. The total score mean (*M*) was 118, median (Mdn) was 121, and standard deviation (SD) was 22.

There were two subtests in the CoMet in which subjects obtained high scores. These subtests were as follows: (2a) writing sentences (min = 0, max = 2, *M* = 1.62, Mdn = 2, SD = .650) and (2b) understanding instructions (min = 0, max = 5, *M* = 4.49, Mdn = 5, SD = 1.188) and (5) drawing task (Picture 1: min = 3, max = 5, *M* = 4.82, Mdn = 5, SD = .479; Picture 2: min = 1, max = 2, *M* = 1.86, Mdn = 2, SD = .352).

**Figure 1:**
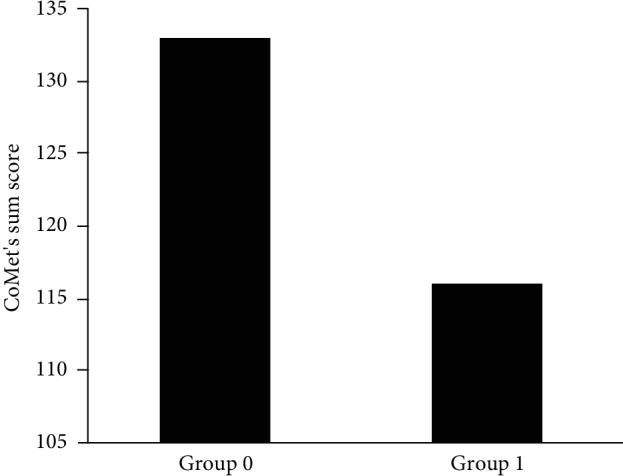
Subjects with neuropsychological dysfunction based on neuropsychologist's clinical judgement (Group 1) achieved lower CoMet total scores.

Based on the clinical neuropsychological assessment, 84% of the patients had at least mild cognitive dysfunction (Group 1). There were twelve patients with minor or no neuropsychological dysfunction (Group 0). We found a significant negative correlation (*r* = ‐.328, *p* = .004) between the CoMet total score and group status (see Figure 1). Cronbach's alpha for all CoMet items was 818, indicating that the parts were 0.818 consistent in a 0 to 1 scale suggesting that the parts have shared covariance and probably measure the same underlying concept. The item-to-total correlations varied between .192 (drawing task, part b) to .812 (episodic memory task).

**Figure 2:**
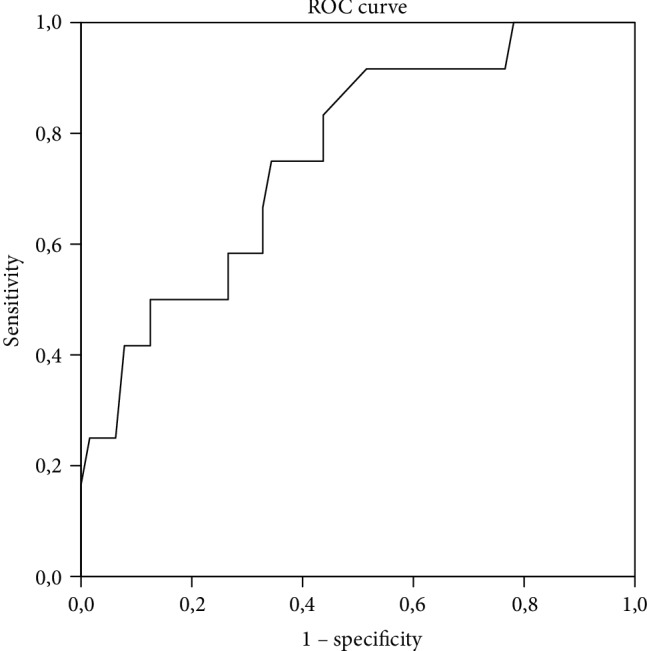
Receiver operating characteristic (ROC) curve for CoMet and cognitive dysfunction.

We performed the nonparametric receiver operating characteristic ROC curve analysis to analyze the sensitivity and specificity of the screening method. Positive predictive value, negative predictive value, sensitivity, and specificity were computed based on two different CoMet total score cut points. Cut points were selectively chosen for sensitivity higher than 70%. Analysis of the receiver operating characteristic ROC curve for the CoMet total score, the cross tabulation of Group 0 and Group 1, and the total sum score revealed that the best balance of sensitivity (88%) and specificity (50%) was at cut point 138. The area under the curve was .76, which means that randomly selected subjects from Group 1 had 76% certainty to be identified as having cognitive dysfunction (see Figure 2). The positive predictive value (PPV) was 90%, and negative predictive value (NPV) was 43%. By adjusting the cut point to 125, the screening method detected 66% of Group 1 cases and specificity was 75%. Self-evaluation was not included in the CoMet total score.

To analyze CoMet's failure to classify correctly, we investigated the false positive and false negative results. False positive results were mainly a consequence of poor memory functioning scores in the CoMet. Subjects who received poor scores for CoMet memory items performed adequately in memory tests from the neuropsychological assessment battery. False negative findings resulted from some subjects who, according to neuropsychological assessment, had mild cognitive dysfunction in working memory, executive function, and/or attention. The CoMet showed these subjects having some deficits in certain tasks, but the total score was so high that these problems did not have adequate weight.

Linear regression analysis showed that age accounted for 10% (beta = ‐.217, *p* = .060) of the sum score variation. Education length also accounted for 10% (beta = .191, *p* = .099) of CoMet total score variation. These sociodemographic variables were independent. The results indicated that there should be one point reduction to the screening method's cut point for every five years of subject's age. Likewise, every ten additional years of education would require a two point increase to cut point. Gender had no effect on CoMet total score variation.

## 4. Discussion

The main aim of the present study was to analyze the validity of the newly developed CoMet in recognizing cognitive deficits after stroke in working-aged patients. The screening method's total score variation was 36–163, indicating that CoMet can be used to differentiate cognitive deficits. Although education had significant impact to CoMet's total score, more than 20 years of education is rather unique. We found a significant correlation between CoMet's total score and the presence of cognitive dysfunction determined on the basis of a clinical judgement of comprehensive neuropsychological assessment batteries. Furthermore, we found a strong internal consistency within the screening. A satisfactory balance of sensitivity and specificity could be identified.

False positive cases resulted from poor memory performance in CoMet in the absence of such deficits in the neuropsychological assessment. It is possible that the CoMet is sensitive to some types of memory deficits not detected in the neuropsychological assessment. Patients with false negative scores seemed to have minor working memory, executive function, and/or attention problems in the neuropsychological assessment, but no such deficits were detected by CoMet. Therefore, the screening method's principles need to be further evaluated to determine if its sensitivity to executive function, working memory, and attention-based tasks can be increased.

CoMet emphasizes visual memory and visual attention more than MMSE or MoCA and is superior in visual memory when compared to the Cambridge Cognition (CAMCOG) [21]. Of the methods used to screen cognitive problems after stroke, MMSE appears to have fairly low sensitivity (62% for cut point value 24) [11]. MoCA's [19] sensitivity (94%) and specificity (42%) were found in <3 weeks poststroke assessment with criterion measure of comprehensive neuropsychological assessment. Subjects' age was, however, higher compared to this study, and 15.7% of patients were observed highly suggestive of dementia. Furthermore, logistic regression selected impairment on MMSE as predictor of impairment on neuropsychological battery over MoCA. Wong et al. [44] found sensitivity (75%) and specificity (95%) with MoCA test among Chinese-speaking participants at 2-4 weeks after aneurysmal subarachnoid hemorrhage and neuropsychological assessment battery as a criterion measure. Results with MMSE were similar. The referenced MoCA validation study did not employ the very restricted inclusion criteria used in the CoMet study, and the study was focused on cognitive impairment with two or more cognitive domain deficits. According to this, the present validation study of CoMet is made with criterion measure of milder cognitive dysfunction. In Smith et al.'s [45] study with older subjects in memory clinic setting (mean age 73.6), MoCA sensitivity for detecting mild cognitive impairment was 83% and specificity 50%. According to this and the fact that MoCA has shown to be insensitive in detecting single domain cognitive deficits [18], the CoMet shows encouraging results for a better ability to differentiate mild cognitive deficit. However, the samples used in previous studies differed considerably from the present sample. Therefore, studies directly comparing CoMet with MoCA and MMSE among the same participants are needed before any firm conclusion can be drawn.

Some patients with moderate and severe dysfunction may have been ruled out from the project with the assumption that they were unlikely to return to work. There is a need for future studies with diverse populations and outcome measures as well evaluating CoMet against neuropsychological assessment in a counterbalanced design. Also, feasibility of administration by medical professionals in different disciplines should be reported in future studies.

It can be concluded that our findings suggest promising results concerning CoMet functionality as a consistent, effortless, and cost-effective way to evaluate poststroke cognitive dysfunction in working-aged patients. Functional cut point score (total score = 138) enables an easy interpretation of the method. In clinical practice, positive screening results should lead to more detailed neuropsychological assessment wherein plans for rehabilitation and future vocational decisions can be made. However, future studies and further development of the method is needed.

## Figures and Tables

**Table 1:** Sociodemographical and etiological information of participants (N77) of this study.

	*N* (%)
Women	21 (27)
Living alone	18 (23)
Vocational school	59 (77)
Occupational status	
Employee	39 (51)
Clerical worker	8 (10)
Superior clerical worker	12 (16)
Entrepreneur	18 (23)
Etiology	
Infarct	56 (73)
ICH	13 (17)
SAV	6 (8)
Other	2 (3)

**Table 2:** Description and scoring for the 12 CoMet subtests.

Subtest	Description	Score range
(1) Orientation	Six orientative questions	0-6
(2a) Writing sentences (2b) Understanding instructions	(a) Writing one sentence from a model and making up another sentence (b) Drawing following five instructions	0-2 0-5
(3) Word fluency	Naming animals in one minute	0-16
(4) Episodic memory	Recalling a story	0-18
(5) Drawing	Drawing two pictures from a model	0-7
(6) Delayed episodic memory	Recalling a story	0-18
(7a) Object naming (7b) Object memory	(a) Naming ten objects from the picture (b) Recalling ten objects	0-10 0-10
(8) Sentence repetition	Repeating a sentence read by the researcher	0-10
(9a) Object replacement (9b) Object recognition	(a) Recalling ten objects and placing them in the right spot (b) Recognizing 10 objects among other objects	0-10 0-10
(10) Visual finding	Finding target symbol among different symbols as quickly as possible	0-18
(11) Number arranging	Arranging twelve numbers in descending order as quickly as possible	0-12
(12) Self-evaluation	Subject's self-evaluation of CoMet performance	N/A

## Data Availability

The data used to support the findings of this study may be released upon application to the Kruunupuisto Rehabilitation center. Contact information: Rehabilitation director Tiina Riikonen Tel: +35850 432 9282. tiina.riikonen@kruunupuisto.fi.

## References

[1] Jaillard A., Naegele B., Trabucco-Miguel S., LeBas J., Hommel M. (2009). Hidden dysfunctioning in subacute stroke. *Stroke*.

[2] Nys G. M. S., van Zandvoort M. J. E., de Kort P. L. M., Jansen B. P. W., de Haan E. H. F., Kappelle L. J. (2007). Cognitive disorders in acute stroke: prevalence and clinical determinants. *Cerebrovascular Diseases*.

[3] Vestling M., Tufvesson B., Iwarsson S. (2003). Indicators for return to work after stroke and the importance of work for subjective well-being and life satisfaction. *Journal of Rehabilitation Medicine*.

[4] Wang Y. C., Kapellusch J., Garg A. (2014). Important factors influencing the return to work after stroke. *Work*.

[5] Danovska M., Peychinska D. (2012). Post-stroke cognitive impairment–phenomenology and prognostic factors. *Journal of IMAB - Annual Proceeding (Scientific Papers)*.

[6] Planton M., Peiffer S., Albucher J. F. (2012). Neuropsychological outcome after a first symptomatic ischaemic stroke with ‘good recovery’. *European Journal of Neurology*.

[7] Viscogliosi C., Desrosiers J., Belleville S., Caron C. D., Ska B., BRAD Group (2011). Differences in participation according to specific cognitive deficits following a stroke. *Applied Neuropsychology*.

[8] Arauz A. (2013). Return to work after stroke: the role of cognitive deficits. *Journal of Neurology, Neurosurgery & PsychiatryJournal of Neurology, Neurosurgery, and Psychiatry*.

[9] de Haan E. H., Nys G. M., Van Zandvoort M. J. (2006). Cognitive function following stroke and vascular cognitive impairment. *Current Opinion in Neurology*.

[10] De Koning I. (2009). Neuropsychological assessment sense and sensibility. *Stroke*.

[11] Kauranen T., Laari S., Turunen K., Mustanoja S., Baumann P., Poutiainen E. (2014). The cognitive burden of stroke emerges even with an intact NIH stroke scale score: a cohort study. *Journal of Neurology, Neurosurgery, and Psychiatry*.

[12] Folstein M. F., Folstein S. E., McHugh P. R. (1975). "Mini-mental state": a practical method for grading the cognitive state of patients for the clinician. *Journal of Psychiatric Research*.

[13] Nys G., Vanzandvoort M., Dekort P., Jansen B., Kappelle L., Dehaan E. (2005). Restrictions of the Mini-Mental State Examination in acute stroke. *Archives of Clinical Neuropsychology*.

[14] Helm-Estabrooks N. (2001). *Cognitive Linguistic Quick Test: CLQT*.

[15] Wechsler D. (2005). *WAIS-III Käsikirja. [The WAIS-III: A Finnish manual]*.

[16] Nasreddine Z. S., Phillips N. A., Bédirian V. (2005). The Montreal Cognitive Assessment, MoCA: a brief screening tool for mild cognitive impairment. *Journal of the American Geriatrics Society*.

[17] Blackburn D. J., Bafadhel L., Randall M., Harkness K. A. (2013). Cognitive screening in the acute stroke setting. *Age and Ageing*.

[18] Pendlebury S. T., Mariz J., Bull L., Mehta Z., Rothwell P. M. (2012). MoCA, ACE-R, and MMSE versus the National Institute of Neurological Disorders and Stroke–Canadian Stroke Network vascular cognitive impairment harmonization standards neuropsychological battery after tia and stroke. *Stroke*.

[19] Godefroy O., Fickl A., Roussel M. (2011). Is the Montreal Cognitive Assessment superior to the Mini-Mental State Examination to detect poststroke cognitive impairment? A study with neuropsychological evaluation. *Stroke*.

[20] Roth M., Huppert F. A., Tym E. (1988). *The Cambridge Examination for Mental Disorders of the Elderly (CAMDEX)*.

[21] Te Winkel-Witlox A. C. M., Post M. W. M., Visser-Meily J. M. A., Lindeman E. (2008). Efficient screening of cognitive dysfunction in stroke patients: comparison between the CAMCOG and the R-CAMCOG, Mini Mental State Examination and Functional Independence Measure-cognition score. *Disability and Rehabilitation*.

[22] Cordell C. B., Borson S., Boustani M. (2013). Alzheimer’s association recommendations for operationalizing the detection of cognitive impairment during the Medicare Annual Wellness Visit in a primary care setting. *Alzheimers Dement*.

[23] Blake H., McKinney M., Treece K., Lee E., Lincoln N. B. (2002). An evaluation of screening measures for cognitive impairment after stroke. *Age and Ageing*.

[24] Stephens S., Kenny R. A., Rowan E. (2004). Neuropsychological characteristics of mild vascular cognitive impairment and dementia after stroke. *International Journal of Geriatric Psychiatry*.

[25] Hochstenbach J., Mulder T., van Limbeek J., Donders R., Schoonderwaldt H. (1998). Cognitive decline following stroke: a comprehensive study of cognitive decline following stroke. *Journal of Clinical and Experimental Neuropsychology*.

[26] Haring H. P. (2002). Cognitive impairment after stroke. *Current Opinion in Neurology*.

[27] Gottesman R. F., Hillis A. E. (2010). Predictors and assessment of cognitive dysfunction resulting from ischaemic stroke. *Lancet Neurology*.

[28] Christensen A. L. (1982). *Lurian Neuropsykologinen Tutkimus. [Luria’s Neuropsychological Investigation. A Finnish Manual]*.

[29] De Renzi E., Faglioni P. (1978). Normative data and screening power of a shortened version of the Token Test. *Cortex*.

[30] Goodglass H., Kaplan E. (1983). *Boston Diagnostic Examination for Aphasia*.

[31] Laine M., Niemi J., Koivuselkä-Sallinen P., Tuomainen J. (1997). *Bostonin Diagnostinen Afasiatutkimus*.

[32] Wechsler D. (2007). *WMS-III Käsikirja. [The WMS-III: A Finnish manual]*.

[33] Benton A. L. (1974). *Revised Visual Retention Test*.

[34] Pulliainen V., Hokkanen L., Salo J., Hänninen T. (1999). *CERAD-Kognitiivinen Tehtäväsarja. Käsikirja. [Manual of the Finnish Version of the CERAD]*.

[35] Vilkki J. (1989). Hemi-inattention in visual search for parallel lines after focal cerebral lesions. *Journal of Clinical and Experimental Neuropsychology*.

[36] Schenkenberg T. (1980). *The Line Bisection Test*.

[37] Nelson H. E. (1976). A modified card sorting test sensitive to frontal lobe defects. *Cortex*.

[38] Delis D. C., Kaplan E., Kramer J. (2001). *Delis-Kaplan Executive Function System*.

[39] Simon H. A. (1975). The functional equivalence of problem solving skills. *Cognitive Psychology*.

[40] Dubois B., Slachevsky A., Litvan I., Pillon B. (2000). The FAB: a frontal assessment battery at bedside. *Neurology*.

[41] Battery A. I. T. (1944). *Manual of Directions and Scoring*.

[42] Van De Loo L. (1956). Enkele beschouwingen over de Bourdon-Wiersma test. *Tijdschr Psychol Kring Nijmeegse Universiteit*.

[43] Golden C. J. (1978). *Stroop Color and Word Test: A Manual for Clinical and Experimental Uses*.

[44] Wong G. K. C., Lam S. W., Wong A., Ngai K., Poon W. S., Mok V. (2013). Comparison of Montreal Cognitive Assessment and Mini-Mental State Examination in evaluating cognitive domain deficit following aneurysmal subarachnoid haemorrhage. *PLoS One*.

[45] Smith T., Gildeh N., Holmes C. (2007). The Montreal Cognitive Assessment: validity and utility in a memory clinic setting. *The Canadian Journal of Psychiatry*.

